# Thawing plateau time indicating the duration of phase transition from ice to water is the strongest predictor for long-term durable pulmonary vein isolation after cryoballoon ablation for atrial fibrillation—Data from the index and repeat procedures

**DOI:** 10.3389/fcvm.2023.1058485

**Published:** 2023-03-06

**Authors:** Li-Bin Shi, Kristian Wollner, Song-Yun Chu, Yu-Chuan Wang, Eivind Solheim, Peter Schuster, Jian Chen

**Affiliations:** ^1^ Department of Clinical Science, University of Bergen, Bergen, Norway; ^2^ Department of Heart Disease, Haukeland University Hospital, Bergen, Norway; ^3^ Department of Cardiology, Peking University First Hospital, Beijing, China; ^4^ Department of Cardiology, Shi Ji Tan Hospital, Beijing, China

**Keywords:** atrial fibrillation, pulmonary vein isolation, cryoballoon, ablation, thawing plateau time

## Abstract

**Introduction:**

This study aimed to clarify the relationship between the durability of pulmonary vein (PV) isolation and the time of phase transition from ice to water indicated by thawing plateau time in a cryoballoon ablation for atrial fibrillation (AF).

**Methods and results:**

In this retrospective study, 241 PVs from 71 patients who underwent a repeat AF ablation 526 (IQR: 412, 675) days after a cryoballoon ablation were analyzed. Reconnection was observed in 101 (41.9%) PVs of 53 patients (74.6%). Thawing plateau time (Time_TP_) was defined as the time from 0°C to 10°C inside the balloon in the thawing period. Durable PV isolation was associated with significantly longer Time_TP_ compared with PV reconnection (26.0 vs. 11.0 s, *P* < 0.001). The proportion of durable PV isolations increased with Time_TP_ in a dose-proportional manner. The cut point for PV reconnection was Time_TP_ <15 s with a positive predictive value of 82.1% (sensitivity = 63.4%, specificity = 90.0%) while for durable PV isolation the cut point was Time_TP_ >25 s with a positive predictive value of 84.6% (sensitivity = 55.0%, specificity = 86.1%). In the analysis of multivariable logistic regression, location of PV reconnection (*P* < 0.01), Time_TP_ (*P* < 0.05) and thawing plateau integral (*P* < 0.01) were shown as independent predictors for durable PV isolation.

**Conclusion:**

Time_TP_ is an independent predictor for the durability of PV isolation, and it presents in a dose-proportional manner. Time_TP_ <15 s predicts long-term reconnection while Time_TP_ >25 s predicts durable PV isolation.

## Introduction

Atrial fibrillation (AF) is one of the most common arrhythmias and electrical isolation of the pulmonary veins (PV) remains the cornerstone of ablation therapy for AF. Recent studies have demonstrated similar clinical outcomes of PV isolation in AF patients achieved by cryoballoon (CB) ablation compared to radiofrequency ablation (1). However, PV electrical reconnection and collateral tissue injuries are still challenges for this technique (2–6). An earlier study has already suggested that cryoablation effect is not only related to freezying temperature, but also other physical factors (7). During CB ablation, both freezing and thawing processes are essential for acute lesion formation. Procedure-related factors determining PV reconnection have also been investigated (8–13). Previous analyses (9, 11) have shown that thawing time, but not freezing time or temperature, impacts PV reconnection, which occurs mostly during long-term follow-up. However, it is unknown whether the entire or only part of the thawing stage plays an important role. During the thawing stage before the CB is deflated, the CB is still adhering to the myocardia of the PV-atrial junction and the blood flow outside the CB is unchanged. Therefore, the temperature in the CB is mostly affected by the temperature of iced tissue around the CB. At the ice melting point the temperature-time curve briefly plateaus, corresponding to the phase transition to water. The duration of the thawing plateau is closely related to the quantity of ice crystals in the myocardia. Thus, we hypothesize that thawing plateau time may reflect the extent of tissue injuries resulting from the freezing/thawing process. We aimed to investigate whether the time of phase transition, as measured by thawing plateau time, might indicate the lesion size and serve as a predictor for durable PV isolation after CB ablation.

## Methods

### Study population

We performed a retrospective analysis in consecutive patients with AF who underwent a repeat ablation procedure in our institute between August 2013 and April 2021. The first-time CB ablation (index procedure) for symptomatic patients with paroxysmal or persistent AF was conducted between April 2012 and July 2020. Patients without complete index procedure data were excluded from the analysis. This study was conducted following the Declaration of Helsinki and approved by the Ethics Committee of Western Norway.

### Index procedure and follow-up

All patients had taken oral anticoagulation for at least 4 weeks and transoesophageal echocardiography was performed before the procedure. The patients underwent the cryoablation procedure under conscious sedation. Heparin was administered immediately after transseptal access to the left atrium. Activated clotting time was kept between 250 and 350 s throughout the procedure. A multiple-electrode diagnostic mapping catheter (Liverwire™, Abbott) was positioned in the coronary sinus and later replaced in the superior caval vein for stimulation of the phrenic nerve.

A steerable 12-Fr sheath (Flexcath®, Medtronic) was placed in the left atrium and angiography of the PVs was performed. A second-generation 28-mm-diameter cryoballoon catheter (Arctic Front Advance®, Medtronic) was introduced into the left atrium through the sheath with a circular mapping catheter (Achieve™, Medtronic) inserted in the lumen. The circular mapping catheter was advanced more distally, if necessary, to support the deployed CB stabilizing at the PV ostium. The PV occlusion after balloon inflation was confirmed by venography. Phrenic nerve stimulation at the high output (up to 20 mA), an inspection of diaphragmatic contraction, and monitoring of the diaphragmatic motor action potential were performed to prevent damage during cryoablation in the right PVs. Freezing duration varied from 180 to 300 s for every single application, with or without an extra application after the PV was isolated at the operator's discretion. Electrical PV isolation was demonstrated by the elimination of all PV ostial potentials recorded by the circular catheter, and the entrance and exit conduction block were proven by stimulation maneuvres.

Plasma level of troponin T (TnT) was determined before and 20–24 h after the index procedure, using an electrochemiluminescence immunoassay on a Modular E system (Roche Diagnostics, Mannheim, Germany). The analytical detection limit was 1 ng/L.

Patients without complications were discharged from the hospital within 1 day of the procedure. Oral anticoagulation was continued for at least 3 months. Patients were followed up in an out-patient clinic with routine ECG, 24-h or 7-day ambulatory electrocardiographic monitoring. Detection of any atrial tachyarrhythmia lasting for longer than 30 s was regarded as a clinical recurrence. Antiarrhythmic drugs were recommended to be discontinued after 3 months if no recurrence of atrial tachyarrhythmia was documented.

### Repeat ablation procedure

Symptomatic patients with AF recurrence were referred to a repeat ablation procedure. All patients were treated with an irrigated radiofrequency ablation catheter with the aid of a 3-dimensional mapping system (EnSite NavX, Abbott, or Carto 3, Biosense Webster, Inc). Procedural details were published (14) earlier. All PVs were checked and reconnection was verified by the recording of the PV potentials. Re-isolation was performed in those reconnected PVs until electrical isolation was achieved.

### Data collection and analysis

**Figure 1:**
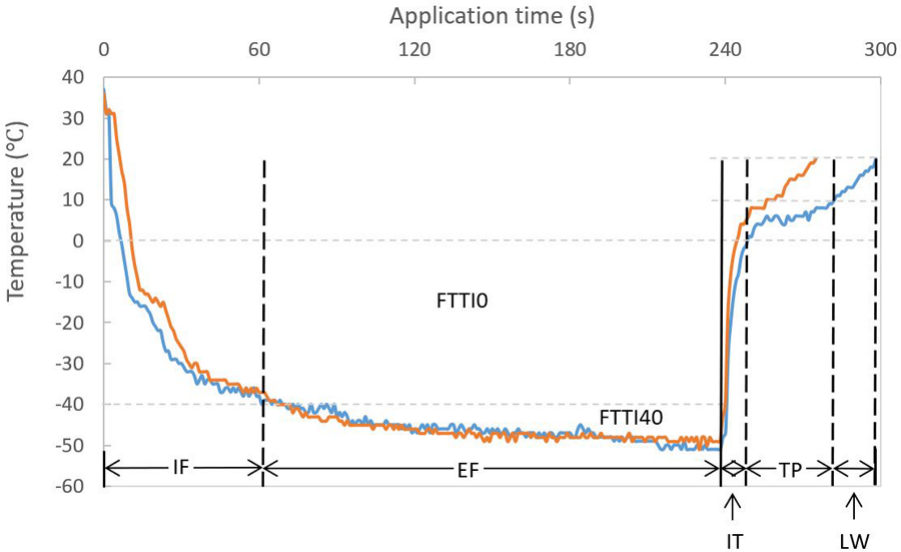
Temperature-time curve during a cryoballoon ablation and descriptions of stages. Similar temperature and time interval during freezing but a short (orange) and long (blue) TP time during thawing are demonstrated. EF, effective freezing; FTTI0, freezing-temperature-time integral under 0°C; FTTI40, freezing-temperature-time integral under −40°C; IF, initial freezing; IT, initial thawing; LW, late warming; TP, thawing plateau. Partition and annotation are based on the blue curve (definition see “Methods”).

All procedural data were recorded during the index procedure and analyzed after the repeat ablation procedure. If more than one freezing was applied in a single PV, the application resulting in eventual PV isolation was included for investigation. All temperature values refer to the inner balloon temperature measured by the thermocouple inside the CB. For further analysis, the freezing stage was divided into two contiguous periods: initial freezing (IF, from start of freezing to temperature decreases to −40°C) and effective freezing (EF, from temperature reaches −40°C to end of freezing). Duration of initial freezing (Time_IF_), duration of effective freezing (Time_EF_), and nadir temperature were recorded. If −40°C was never achieved, Time_IF_ was defined as the time from the start of freezing to the point of the nadir temperature. Freezing-temperature-time integrals under 0°C (FTTI0) and −40°C (FTTI40) were defined as the areas bounded by the temperature curve and 0°C and −40°C, respectively. The thawing stage was divided into three contiguous periods: initial thawing (IT, from the end of freezing to temperature reaches 0°C), thawing plateau (TP, from 0°C to 10°C) and late warming (LW, from 10°C to 20°C when CB deflated). Duration of the corresponding stages (Time_IT_, Time_TP_, and Time_LW_) were recorded. Initial thawing integral, thawing plateau integral and late warming integral were defined as the areas bounded by the temperature curve and 0°C, 10°C and 20°C for IT, TP and LW, respectively. Exemplified temperature-time curves during the cryoablation and the specified measurements are shown in Figure 1. The accumulated values of these parameters (referred to below) are the corresponding sums for all PVs.

### Statistical analysis

Continuous variables were presented as mean ± standard deviation if normally distributed, or as median and interquartile ranges (IQR) if not normally distributed, after the Shapiro-Wilk test. To compare means of continuous data, a 2-sample *t*-test and Mann-Whitney *U* test were employed for parametric and nonparametric analysis. Categorical values were presented as percentages and analyzed using the *χ*^2^ test or Fisher's exact test as appropriate. Logistic regression analysis was performed to evaluate the effects of the procedural parameters on the presence of PV reconnection. Variables with a *P*-value over 0.1 in the univariate analysis were removed from the model for further analysis. The correlation was tested among continuous variables, using Pearson's correlation coefficient for normally distributed data. Otherwise, Spearman's correlation was applied as appropriate. Statistical analysis was performed with SPSS version 26 (IBM, USA). A *P*-value <0.05 was considered statistically significant.

## Results

### Characteristics of the study population, ablation procedures and the pulmonary veins

A total of 71 patients with recurrent AF were enrolled in this study. The baseline characteristics of patients and information of index procedures are shown in Table 1. The common left pulmonary vein (LPV) was observed in 12 patients. Data were missed or applications were interrupted by technical problems in 6 PVs and cryoablation failed in index procedure in 4 PVs. A total of 21 PVs were excluded in which more than 3 applications were applied, to avoid the accumulative point-by-point freezing effect. Finally, 241 PVs were analyzed. Reconnection was observed in 101 (41.9%) PVs of 53 patients (74.6%). No extra application was performed in 168 PVs (69.7%). Complete occlusion was achieved in 173 PVs (71.8%), and suboptimal occlusion with minor leakage in 68 (28.2%). Successful isolation was achieved after the first freezing in 184 PVs (76.3%).

**Table 1:** Baseline characteristics of the patients and index cryoablation procedures.

Characteristic (*n* = 71)	Value
Age (year)	61.8 ± 10.5
Male, *n* (%)	43 (60.6)
Body mass index (kg/m^2^)	27.7 ± 4.8
Hypertension, *n* (%)	32 (45.1)
Diabetes mellitus, *n* (%)	2 (2.8)
Coronary heart disease, *n* (%)	7 (9.9)
Paroxysmal atrial fibrillation, *n* (%)	42 (59.2)
Information on index procedure	
Accumulated time of freezing (minute)	25.0 (17.0, 32.0)
Fluoroscopy time (minute)	14.0 (10.0, 20.0)
Procedure time (minute)	90.0 (70.0, 120.0)
Duration from index to repeat procedure (day)	526 (412, 675)

### Relationship between the procedural parameters and the durability of PV isolation

**Figure 2:**
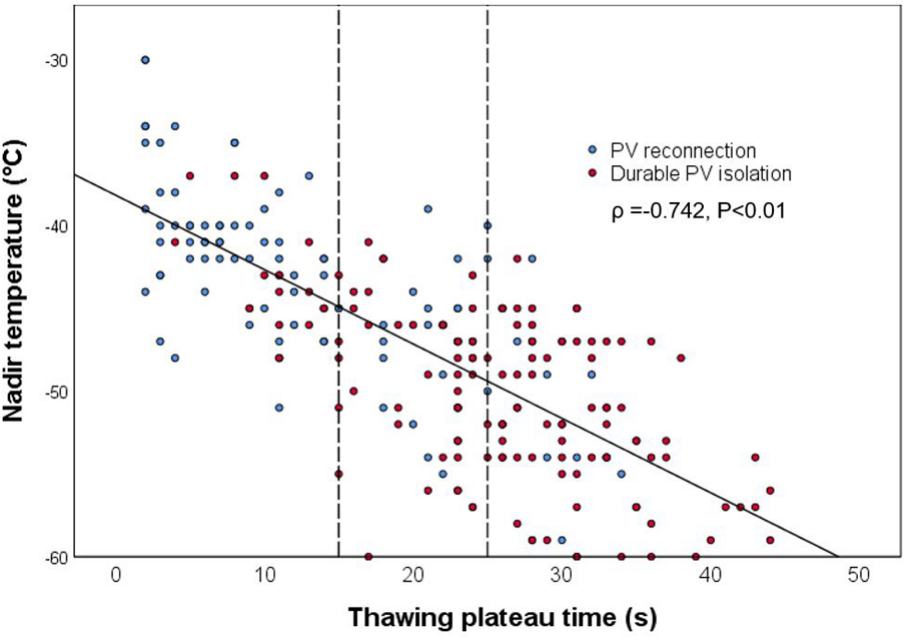
Correlation between nadir temperature and thawing plateau time. The solid line shows the trend of correlation, while the dotted lines demonstrate cut points for PV reconnection (15 s) and for durable PV isolation (25 s), respectively. PV, pulmonary vein.

The mean Time_TP_ in this cohort was 23.0 (11.0, 28.0) s, and it correlated with the nadir temperature (*ρ *= −0.742; *P* < 0.01; Figure 2). The possible procedural and biophysical parameters associated with PV reconnection during long-term follow-up are listed and compared between groups in Table 2. Besides longer Time_TP_ (*P* < 0.01), those cryoablations leading to durable PV isolation showed lower nadir temperature (*P* < 0.01), shorter Time_IF_ and longer Time_EF_ (*P* < 0.01), higher FTTI0 and FTTI40 (*P* < 0.01), longer total thawing time and Time_IT_ (*P* < 0.01), and higher initial thawing integral and thawing plateau integral (*P* < 0.01. Univariate analysis in Table 2). In the analysis of multivariable logistic regression, location of PV reconnection (most notably the left superior PV, *P* < 0.01), Time_TP_ (*P* < 0.05) and thawing plateau integral (*P* < 0.01) were shown as independent predictors for durable PV isolation (Table 2).

**Table 2:** Association between procedural parameters and durable PV isolation.

Factors	Durable PV isolation		Univariate	Multivariable	
	No (*n* = 102)	Yes (*n* = 140)	*P* value	*P* value	OR (95% CI)
**Location of the reconnection, *n* (%)**			0.002	0.005	
Left common PV	5 (55.6%)	4 (44.4%)	0.906		
Left superior PV	14 (24.6%)	43 (75.4%)**	0.002	0.005	4.36 (1.57, 12.07)
Left inferior PV	17 (31.5%)	37 (68.5%)*	0.020		
Right superior PV	34 (54.0%)	29 (46.0%)	0.954		
Right inferior PV	31 (53.4%)	27 (44.6%)	1.000		
Time to isolation (s) (*n* = 43 and 85)	50.0 (39.0, 60.0)	48.0 (33.5, 70.0)	†		
Total application number	1 (1, 2)	1 (1, 2)	0.939		
No extra application, *n* (%) (*n* = 168)	71 (42.2%)	97 (57.8%)	0.676		
PV isolation at the first application, *n* (%) (*n* = 184)	75 (40.8%)	109 (59.2%)	0.226		
**Freezing stage**					
Nadir temperature (°C)	−43.9 ± 5.6	−50.2 ± 5.4**	0.000		
Total freezing duration (s)	240.1 ± 35.0	228.8 ± 37.6*	0.005		
Time_IF_ (s)	78.0 (56.0, 125.0)	54.5 (42.0, 74.0)**	0.000		
Time_EF_ (s)	158.0 (110.5, 184.0)	170.5 (141.0, 191.0)**	0.002		
FTTI0 (×10^3^, °C·s)	8.6 (8.0, 9.6)	9.2 (8.4, 10.3)**	0.002		
FTTI40 (×10^3^, °C·s)	0.3 (0, 0.9)	1.2 (0.6, 1.7)**	0.000		
**Thawing stage**					
Total thawing time (s)	32.0 (21.0, 45.5)	50.0 (41.0, 60.0)**	0.000		
Time_IT_ (s)	5.0 (4.0, 7.5)	9.0 (7.3, 12.0)**	0.000	0.052	
Time_TP_ (s)	11.0 (6.0, 22.5)	26.0 (22.0, 31.0)**	0.000	0.025	1.08 (1.01, 1.16)
Time_LW_ (s)	13.0 (9.0, 18.5)	16.0 (12.0, 20.0)*	0.126		
Initial thawing integral (°C·s)	101.0 (79.5, 140.0)	171.0 (136.3, 214.5)**	0.000		
Thawing plateau integral (°C·s)	37.0 (17.0, 83.5)	127.5 (86.3, 168.5)**	0.000	0.003	1.02 (1.01, 1.03)
Late warming integral (°C·s)	59.0 (41.0, 85.0)	72.5 (58.0, 88.0)**	0.138		

EF, effective freezing; FTTI0, freezing-temperature-time integral under 0°C; FTTI40, freezing-temperature-time integral under −40°C; IF, initial freezing; IT, initial thawing; LW, late warming; PV, pulmonary vein; TP, thawing plateau.

* *P* < 0.05, compared with the group without durable PV isolation.

** *P* < 0.01, compared with the group without durable PV isolation.

^†^ Not involved in regression analysis because of fewer observation cases.

**Figure 3:**
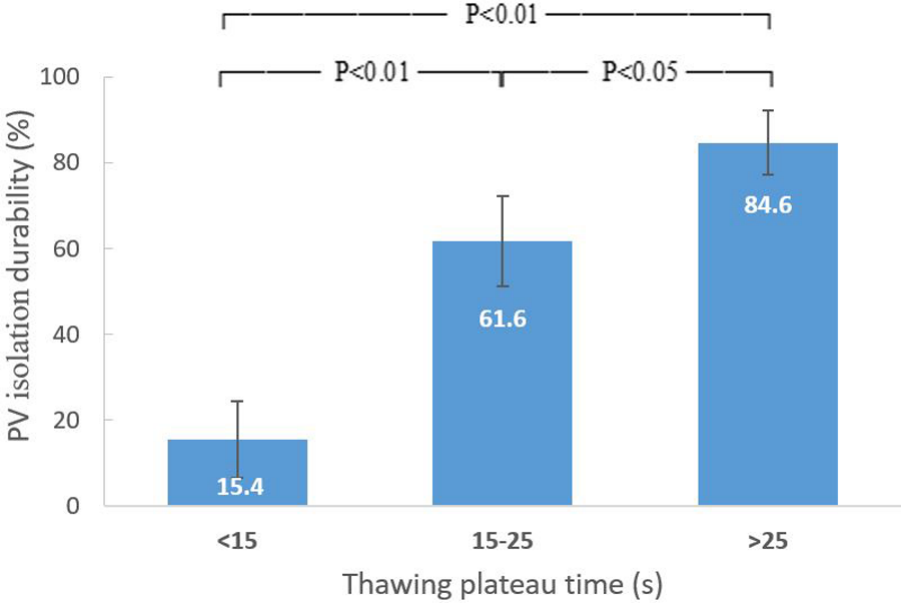
Relation between thawing plateau time and durability of PV isolation. The durability of PV isolation increased with thawing plateau time in a dose-proportional manner. PV, pulmonary vein.

The durability of PV isolation increased with Time_TP_ in a dose-proportional manner (Figure 3). The cut point for PV reconnection was Time_TP_ <15 s with a positive predictive value of 82.1% (sensitivity = 63.4%, specificity = 90.0%), while for durable PV isolation the cut point was Time_TP_ >25 s with a positive predictive value of 84.6% (sensitivity = 55.0%, specificity = 86.1%). For those applications with a Time_TP_ between 15 and 25 s, nadir temperature lower than −45°C was employed as a supplementary predictor for durable PV isolation with a positive predictive value of 78.4% (sensitivity = 59.2%, specificity = 68.0%).

**Figure 4:**
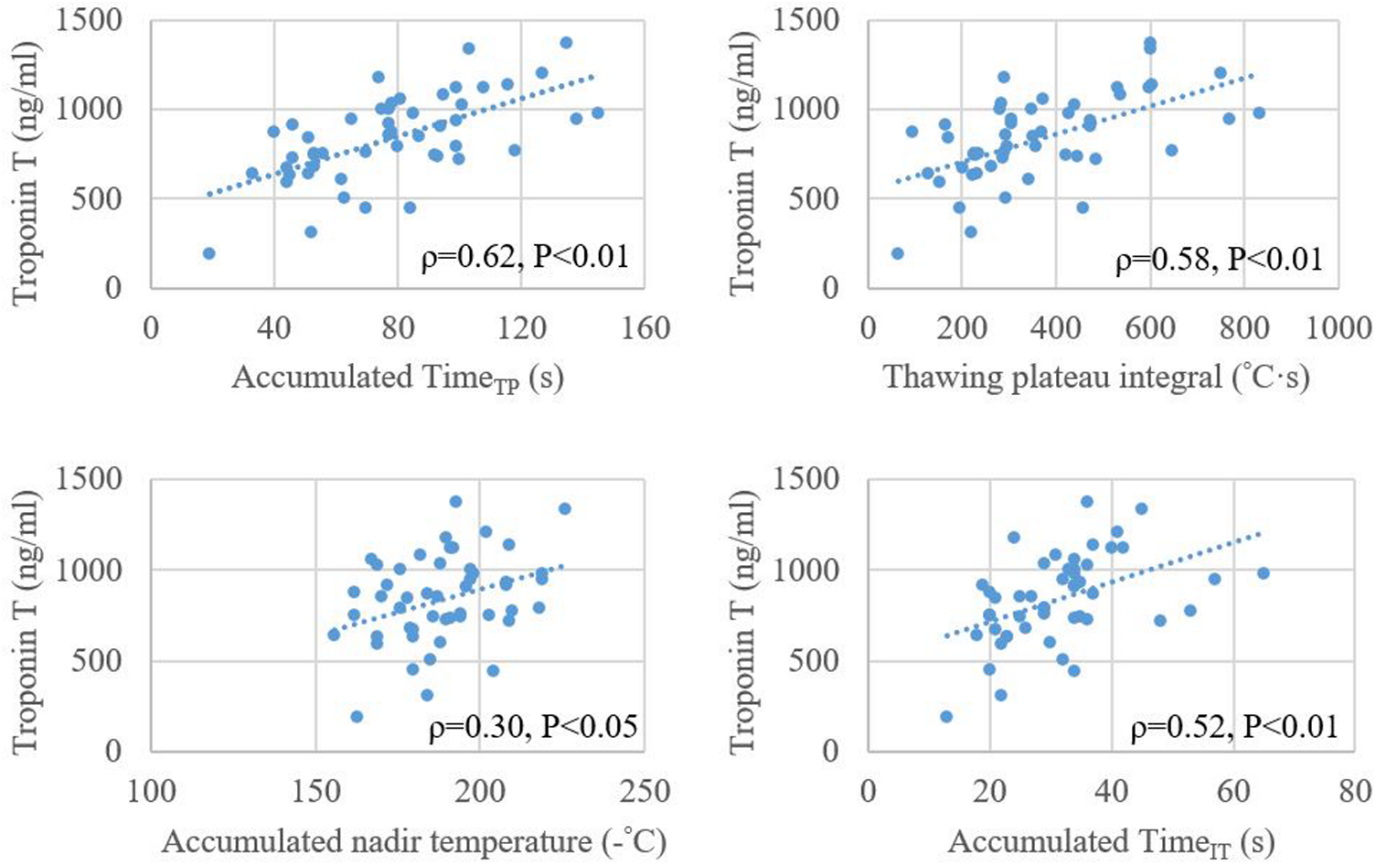
Relationship between plasma level of troponin T after cryoballoon ablation and accumulated values of time_TP_, thawing plateau integral, nadir temperature, and time_IT_. Time_IT_, initial thawing time; Time_TP_, thawing plateau time.

The average plasma TnT after the index procedure was 835.3 ± 244.1 ng/L. The correlation between the accumulated Time_TP_ and TnT level (*ρ* = 0.624, *P* < 0.01) was stronger than the accumulated values of thawing plateau integral, Time_IT_ and nadir temperature (Figure 4).

## Discussion

In this retrospective study, we demonstrated that the durability of PV isolation increased with longer Time_TP_ during the thawing stage in a dose-proportional manner. The accumulated Time_TP_ positively correlated with TnT level after cryoablation.

### The thawing plateau reflects the mass of the frozen tissue

Injury to cells during the freezing process is associated with extracellular and intracellular ice formation and the development of an osmotic gradient between the cells and interstitium. If lower temperatures are reached (−20°C to −40°C), recrystallization may take place during thawing with further structural destruction (15). The freeze and thaw cycle is important for lesion formation. However, previous studies have shown that biophysical parameters in the thawing stage, but not freezing duration or nadir temperature, may predict PV reconnection (9, 11). Frozen tissue around the CB forms an “ice cap” after the delivery of pressurized cryorefrigerant (nitrous oxide). During the thawing period, the flow of refrigerant has been interrupted and the balloon temperature measured by the thermocouple rises, warmed by the blood flow. When the temperature reaches 0°C the ice cap starts to melt. At this point the temperature-time curve exhibits a plateau corresponding to the ice-water phase transition. Energy that would otherwise raise the temperature of the ice-cap, instead provides the latent heat of fusion required to melt the ice. The greater the mass of the frozen tissue, the more energy is required and the longer the time of phase transition. The thawing plateau time therefore serves as a surrogate for the phase transition time. Furthermore, it represents the mass of the ice cap that forms during freezing, which is the critical factor for lesion formation.

### The predictive value of time_TP_ for the durability of PV isolation

The thawing time has been suggested as a predictor for the durability of PV isolation. Ghosh et al. reported that a thawing time of ≥25 s between −30°C and +15°C may predict permanent PV isolation (9). Aryana et al. indicated a thawing time (defined as time to reach 0°C, the same as Time_IT_ in our study) of ≥10 s may predict PV isolation durability (11). Our findings further support their arguments. More importantly, we have demonstrated that the most decisive part in the thawing period is the thawing plateau and have shown that Time_TP_ is linked to the durability of PV isolation in a dose-proportional manner. We found that Time_TP_ <15 and >25 s can predict 82.1% of reconnection and 84.6% of durable PV isolation, respectively. Multivariable regression analysis showed Time_TP_ to be the strongest biophysical predictor among others that were significant in the univariate investigation. This might be explained by the following facts and observations. First, Time_TP_ represents the amount of frozen tissue created by the CB ablation, as abovementioned. Second, the balloon temperature at the thawing plateau is much closer to the tissue temperature than at other stages (16). Third, recrystallization frequently takes place in this stage and even prolongs the duration (10, 17). Finally, the rate of thawing has a significant effect on osmotic stress, and slow thawing, in general, is more likely to cause fluid shifts and increase osmotic trauma to the cells (10, 17). This hypothesis was further supported by the correlation between Time_TP_ and TnT. Although TnT is not a predictor for the clinical outcome of CB ablation for AF, it precisely quantifies the magnitude of myocardial injury (18). In the present study, accumulated Time_TP_ positively correlated with the elevation of TnT after the index procedure, which suggested Time_TP_ may serve as a parameter to estimate lesion size. From clinical perspective, the time between 0°C and 10°C is automatically recorded by the system, and it is easy to read out and can be potentially displayed after an application software is developed in the future.

Our results indicate that after a CB ablation with the elimination of the PV potentials, if the Time_TP_ <15 s, an extra application is probably necessary; if the Time_TP_ >25 s, no further application is needed. In the “grey zone” between 15 and 25 s, it is difficult to find a cutoff value. Combined with the nadir temperature not reaching −45°C, additional applications should be considered.

Time-to-isolation (TTI) has been proven as a predictor for the durability of PV isolation, and several patient-tailored dosing strategies based on TTI showed similar clinical outcomes with the conventional 240-second procedure (19, 20). We did not analyze the relationship between TTI and the durability of PV isolation in this study. This was due to the unavailability of TTI in many cases, because the operators tended to advance the circular catheter further distal into the PVs to support a stable PV occlusion during freezing.

The CB temperature during freezing has also been studied. In an animal study, a rapid temperature drop to below −40°C associated with maximal tissue damage (17). Fürnkranz et al. reported an association between nadir temperature at −51°C and acute PV isolation (8). Unfortunately, the predictive value of the nadir temperature during freezing for the durability of PV isolation has not been confirmed by clinical trials. Our data were consistent with the study by Aryana et al. showing that the nadir temperature in the durable PV isolation group was lower than that in the reconnection group, but it was not an independent predictor for durable PV isolation. Ciconte et al. reported the achievement of −40°C within 60 s as an independent predictor for durable PV isolation (21). Later, a similar finding was demonstrated in a propensity score-matched case-control study, comparing a dosing strategy based on achieving −40°C within 60 s with a real-time signal guiding approach (22). In the present study, we evaluated the time below −40°C and FTTI40 which combined the impacts of time and temperature. There was a difference between the reconnection group and the durable group, but neither was an independent predictor. A possible explanation for these findings might be that the balloon temperature does not accurately reflect the tissue temperature during freezing, due to the remote location of the thermocouple. Another could be the interference of delivery of pressurized cryorefrigerant. As shown in Figure 1, two different thawing patterns are demonstrated following the same freezing profile. The finding that the left superior PV was related to higher durability of PV isolation was probably due to simple anatomy, a more favorable fitting angle making it easier to achieve optimal CB ablation. In contrast, the left common and the right PVs were associated with worse outcomes.

### Limitations

First, to be accurate, Time_TP_ should be measured by the precise interval of the thawing plateau. However, the beginning and end of the plateau on the curve are difficult to define in practice and variation exists even for a single observer. We employed the thawing time between 0°C and 10°C to standardize and simplify the measurement. Since the plateau always lies in this narrow range, the difference is little and acceptable. Second, Time_TP_ is a parameter that is acquired after an application, it cannot be used for adjusting the duration during the application. Third, the impacts of TTI and freezing dosage/duration were not analyzed because of the unavailability of data or identical regimen. Fourth, variations of anatomical and pathophysiological features, including the size and angle of the PVs, and mitral regurgitation, may influence freezing and thawing to some extent. We could not conduct subgroup analysis of a single PV because of the limited sample size. Finally, as there was no major complication observed among study patients, we could not verify the predictive value of Time_TP_ for complications.

## Conclusion

Time_TP_ is an independent predictor for the durability of PV isolation, and it presents in a dose-proportional manner. Time_TP_ <15 s predicts long-term reconnection while Time_TP_ >25 s predicts durable PV isolation. These findings may guide the regulation of further cryoballoon ablation regimens.

## Data Availability

The original contributions presented in the study are included in the article/Supplementary Material, further inquiries can be directed to the corresponding author.

## References

[1] Kuck KH Brugada J Furnkranz A Metzner A Ouyang F Chun KR Cryoballoon or radiofrequency ablation for paroxysmal atrial fibrillation. N Engl J Med. (2016) 374:2235–45. 10.1056/NEJMoa1602014 27042964

[2] Buist TJ Adiyaman A Smit JJJ Ramdat Misier AR Elvan A. Arrhythmia-free survival and pulmonary vein reconnection patterns after second-generation cryoballoon and contact-force radiofrequency pulmonary vein isolation. Clin Res Cardiol. (2018) 107:498–506. 10.1007/s00392-018-1211-9 29411114

[3] Shigeta T Okishige K Aoyagi H Nishimura T Nakamura RA Ito N Clinical investigation of esophageal injury from cryoballoon ablation of persistent atrial fibrillation. Pacing Clin Electrophysiol. (2019) 42:230–7. 10.1111/pace.13629 30549044

[4] Bellmann B Hubner RH Lin T Paland M Steiner F Krause P Bronchial injury after atrial fibrillation ablation using the second-generation cryoballoon. Circ Arrhythm Electrophysiol. (2018) 11:e005925. 10.1161/CIRCEP.117.005925 29874170

[5] Abugattas JP de Asmundis C Iacopino S Salghetti F Takarada K Coutino HE Phrenic nerve injury during right inferior pulmonary vein ablation with the second-generation cryoballoon: clinical, procedural, and anatomical characteristics. Europace. (2018) 20:e156–63. 10.1093/europace/eux337 29182748

[6] Heeger CH Sohns C Pott A Metzner A Inaba O Straube F Phrenic nerve injury during cryoballoon-based pulmonary vein isolation: results of the worldwide YETI registry. Circ Arrhythm Electrophysiol. (2022) 15:e010516. 10.1161/CIRCEP.121.010516 34962134 PMC8772436

[7] Theuns DA Kimman GP Szili-Torok T Res JC Jordaens LJ. Ice mapping during cryothermal ablation of accessory pathways in WPW: the role of the temperature time constant. Europace. (2004) 6:116–22. 10.1016/j.eupc.2003.11.006 15018869

[8] Furnkranz A Koster I Chun KR Metzner A Mathew S Konstantinidou M Cryoballoon temperature predicts acute pulmonary vein isolation. Heart Rhythm. (2011) 8:821–5. 10.1016/j.hrthm.2011.01.044 21315836

[9] Ghosh J Martin A Keech AC Chan KH Gomes S Singarayar S Balloon warming time is the strongest predictor of late pulmonary vein electrical reconnection following cryoballoon ablation for atrial fibrillation. Heart Rhythm. (2013) 10:1311–7. 10.1016/j.hrthm.2013.06.014 23792110

[10] Knecht S Kuhne M Osswald S Sticherling C. Quantitative assessment of a second-generation cryoballoon ablation catheter with new cooling technology-a perspective on potential implications on outcome. J Interv Card Electrophysiol. (2014) 40:17–21. 10.1007/s10840-014-9883-1 24622931

[11] Aryana A Mugnai G Singh SM Pujara DK de Asmundis C Singh SK Procedural and biophysical indicators of durable pulmonary vein isolation during cryoballoon ablation of atrial fibrillation. Heart Rhythm. (2016) 13:424–32. 10.1016/j.hrthm.2015.10.033 26520204

[12] Deubner N Greiss H Akkaya E Zaltsberg S Hain A Berkowitsch A The slope of the initial temperature drop predicts acute pulmonary vein isolation using the second-generation cryoballoon. Europace. (2017) 19:1470–7. 10.1093/europace/euw192 27702863

[13] Chen S Schmidt B Bordignon S Perrotta L Bologna F Chun KRJ. Impact of cryoballoon freeze duration on long-term durability of pulmonary vein isolation: ICE re-map study. JACC Clin Electrophysiol. (2019) 5:551–9. 10.1016/j.jacep.2019.03.012 31122376

[14] Shi LB Rossvoll O Tande P Schuster P Solheim E Chen J. Cryoballoon vs. radiofrequency catheter ablation: insights from Norwegian randomized study of PERSistent atrial fibrillation (NO-PERSAF study). Europace. (2022) 24:226–33. 10.1093/europace/euab281 35134151 PMC8824490

[15] Kaszala K Ellenbogen KA. Biophysics of the second-generation cryoballoon: cryobiology of the big freeze. Circ Arrhythm Electrophysiol. (2015) 8:15–7. 10.1161/CIRCEP.115.002675 25691553

[16] Takami M Misiri J Lehmann HI Parker KD Johnson SB Sarmiento RI Spatial and time-course thermodynamics during pulmonary vein isolation using the second-generation cryoballoon in a canine in vivo model. Circ Arrhythm Electrophysiol. (2015) 8:186–92. 10.1161/CIRCEP.114.002137 25532529

[17] Gage AA Baust J. Mechanisms of tissue injury in cryosurgery. Cryobiology. (1998) 37:171–86. 10.1006/cryo.1998.2115 9787063

[18] Gulati M Levy PD Mukherjee D Amsterdam E Bhatt DL Birtcher KK 2021 AHA/ACC/ASE/CHEST/SAEM/SCCT/SCMR guideline for the evaluation and diagnosis of chest pain: executive summary: a report of the American college of cardiology/American heart association joint committee on clinical practice guidelines. Circulation. (2021) 144:e368–454. 10.1161/CIR.0000000000001029 34709928

[19] Aryana A Kenigsberg DN Kowalski M Koo CH Lim HW O'Neill PG Verification of a novel atrial fibrillation cryoablation dosing algorithm guided by time-to-pulmonary vein isolation: results from the cryo-DOSING study (cryoballoon-ablation DOSING based on the assessment of time-to-effect and pulmonary vein isolation guidance). Heart Rhythm. (2017) 14:1319–25. 10.1016/j.hrthm.2017.06.020 28625929

[20] Chun KR Stich M Furnkranz A Bordignon S Perrotta L Dugo D Individualized cryoballoon energy pulmonary vein isolation guided by real-time pulmonary vein recordings, the randomized ICE-T trial. Heart Rhythm. (2017) 14:495–500. 10.1016/j.hrthm.2016.12.014 27956248

[21] Ciconte G Mugnai G Sieira J Velagic V Saitoh Y Irfan G On the quest for the best freeze: predictors of late pulmonary vein reconnections after second-generation cryoballoon ablation. Circ Arrhythm Electrophysiol. (2015) 8:1359–65. 10.1161/CIRCEP.115.002966 26527624

[22] Iacopino S Mugnai G Takarada K Paparella G Stroker E De Regibus V Second-generation cryoballoon ablation without the use of real-time recordings: a novel strategy based on a temperature-guided approach to ablation. Heart Rhythm. (2017) 14:322–8. 10.1016/j.hrthm.2016.11.023 27871986

